# Age is not just a number: Naive T cells increase their ability to persist in the circulation over time

**DOI:** 10.1371/journal.pbio.2003949

**Published:** 2018-04-11

**Authors:** Sanket Rane, Thea Hogan, Benedict Seddon, Andrew J. Yates

**Affiliations:** 1 Department of Pathology and Cell Biology, Columbia University Medical Center, New York, New York, United States of America; 2 Institute of Infection, Immunity & Inflammation, College of Medical, Veterinary & Life Sciences, University of Glasgow, Glasgow, United Kingdom; 3 Institute of Immunity and Transplantation, Division of Infection and Immunity, UCL, Royal Free Hospital, London, United Kingdom; University of Pennsylvania Perelman School of Medicine, United States of America

## Abstract

The processes regulating peripheral naive T-cell numbers and clonal diversity remain poorly understood. Conceptually, homeostatic mechanisms must fall into the broad categories of neutral (simple random birth–death models), competition (regulation of cell numbers through quorum-sensing, perhaps via limiting shared resources), adaptation (involving cell-intrinsic changes in homeostatic fitness, defined as net growth rate over time), or selection (involving the loss or outgrowth of cell populations deriving from intercellular variation in fitness). There may also be stably maintained heterogeneity within the naive T-cell pool. To distinguish between these mechanisms, we confront very general models of these processes with an array of experimental data, both new and published. While reduced competition for homeostatic stimuli may impact cell survival or proliferation in neonates or under moderate to severe lymphopenia, we show that the only mechanism capable of explaining multiple, independent experimental studies of naive CD4^+^ and CD8^+^ T-cell homeostasis in mice from young adulthood into old age is one of adaptation, in which cells act independently and accrue a survival or proliferative advantage continuously with their post-thymic age. However, aged naive T cells may also be functionally impaired, and so the accumulation of older cells via ‘conditioning through experience’ may contribute to reduced immune responsiveness in the elderly.

## Author summary

The body maintains large populations of naive T cells, a type of white blood cell that is able to respond specifically to pathogens. This arsenal is essential for our capacity to fight novel infections throughout our lifespan, and their numbers remain quite stable despite a gradual decline in the production of new naive T cells as we age. However, the mechanisms that underlie this stability are not well understood. In this study, we address this problem by testing a variety of potential mechanisms, each framed as a mathematical model, against multiple datasets obtained from experiments performed in mice. Our analysis supports a mechanism by which naïve T cells gradually increase their ability to survive the longer they reside in the circulation. Paradoxically, however, naïve T cells may also lose their ability to respond effectively to infections as they age. Together, these processes may drive the accumulation of older, functionally impaired T cells, potentially at the expense of younger and more immunologically potent cells, as we age.

## Introduction

Naive T cells accumulate in the periphery rapidly from birth, but their numbers decline gradually from puberty onwards in both mice and humans due to the slow involution of thymus and associated decline in the export of new cells [1, 2]. Despite substantial knowledge of the qualitative nature of the cues involved in their survival and proliferative renewal—which include signals through the T-cell receptor (TCR) and from cytokines—we have a relatively limited quantitative understanding of how the total numbers and receptor diversity of naive T cells are determined throughout life.

The consensus in the field has been that the population dynamics of naive T cells are influenced by intra- and/or interclonal competition for limiting homeostatic cues, largely motivated by observations that homeostatic proliferation and cell longevity increase under severely lymphopenic conditions [3–7]. In support of this hypothesis, mathematical models of resource competition—in which all cells compete for a limiting, ‘public’ supply of homeostatic stimuli—have successfully described naive T-cell population dynamics in lymphoreplete and partially lymphopenic settings [8, 9]. However, multiple observations indicate that these models have limited explanatory power. The extent to which resource competition, or any similar quorum-sensing mechanism, influences cell lifetimes or division rates under replete conditions is unclear [10–12], and resource competition alone is unable to explain the kinetics of replacement of old naive T cells by new cells exported from the thymus in healthy mice [13]. There is also evidence that naive T cells’ homeostatic fitness, defined as the difference between their rates of division and loss, may vary with host or cell age. Naive TCR transgenic T cells from aged mice persist longer than the same cells from young mice following transfer, and naive T cells are lost more slowly following thymectomy in old mice than in young mice [14].

There are at least two mechanisms that may generate heterogeneity in homeostatic fitness and potentially explain these observations. One is a process of adaptation, in which cells accumulate changes, possibly in response to microenvironmental signals, that improve survival or the ability to self-renew through division the longer they survive [13, 15]. Such changes might, for example, reflect the continued maturation of recent thymic emigrants (RTEs) in the periphery [16, 17]. Another mechanism is a process of selection acting upon cell populations exhibiting a distribution of stable, cell-intrinsic rates of homeostatic division or loss [18–20]. This variation in fitness might derive from clone-specific differences in TCR affinity for self-peptide–MHC ligands [21] or potentially levels of expression of the interleukin 7 (IL-7) receptor [22], although to our knowledge the impact of the latter is manifest only in lymphopenia [23]. Modelling has quantified the extent to which heterogeneity in naive cells’ capacity to survive can select for intrinsically long-lived cells over time [13, 19, 24]. Crucially, however, adaptive and selective mechanisms are difficult to distinguish directly using standard cross-sectional studies of T-cell dynamics in mice because they can give rise to qualitatively similar distributions of fitnesses at the cell population level.

Mathematical models are appropriate tools for describing cell population dynamics, and when combined with experimental data, they can boost our ability to discriminate between candidate immunological mechanisms. Furthermore, a model’s worth can be assessed both by its ability to explain a particular dataset (its goodness of fit) as well as its ability to successfully describe multiple independent experiments (its robustness). With this philosophy in mind, we confronted models of different homeostatic mechanisms with a diverse set of data, generated by ourselves and from other laboratories. Specifically, we compared different models’ abilities to explain (i) naive T-cell dynamics in healthy, replete mice and following thymectomy, (ii) the dynamics of infiltration of new naive cells from the thymus into an intact peripheral compartment, and (iii) cotransfers of cells of different ages into the same host. Together, these data allowed us to rule out purely homogeneous models in which all naive T cells compete equally for homeostatic stimuli. Furthermore, we found that a model of cellular adaptation, in which a cell’s fitness increases with its post-thymic age, was uniquely able to explain all three disparate datasets. While adaptation, selection, and quorum-sensing are not mutually exclusive and may operate in combination, we argue that, under normal physiological circumstances, adaptation is the dominant force shaping naive T-cell population dynamics because in isolation it provides a parsimonious explanation of diverse experimental data.

## Materials and methods

### Describing candidate mechanisms of naive T-cell population dynamics

**Fig 1:**
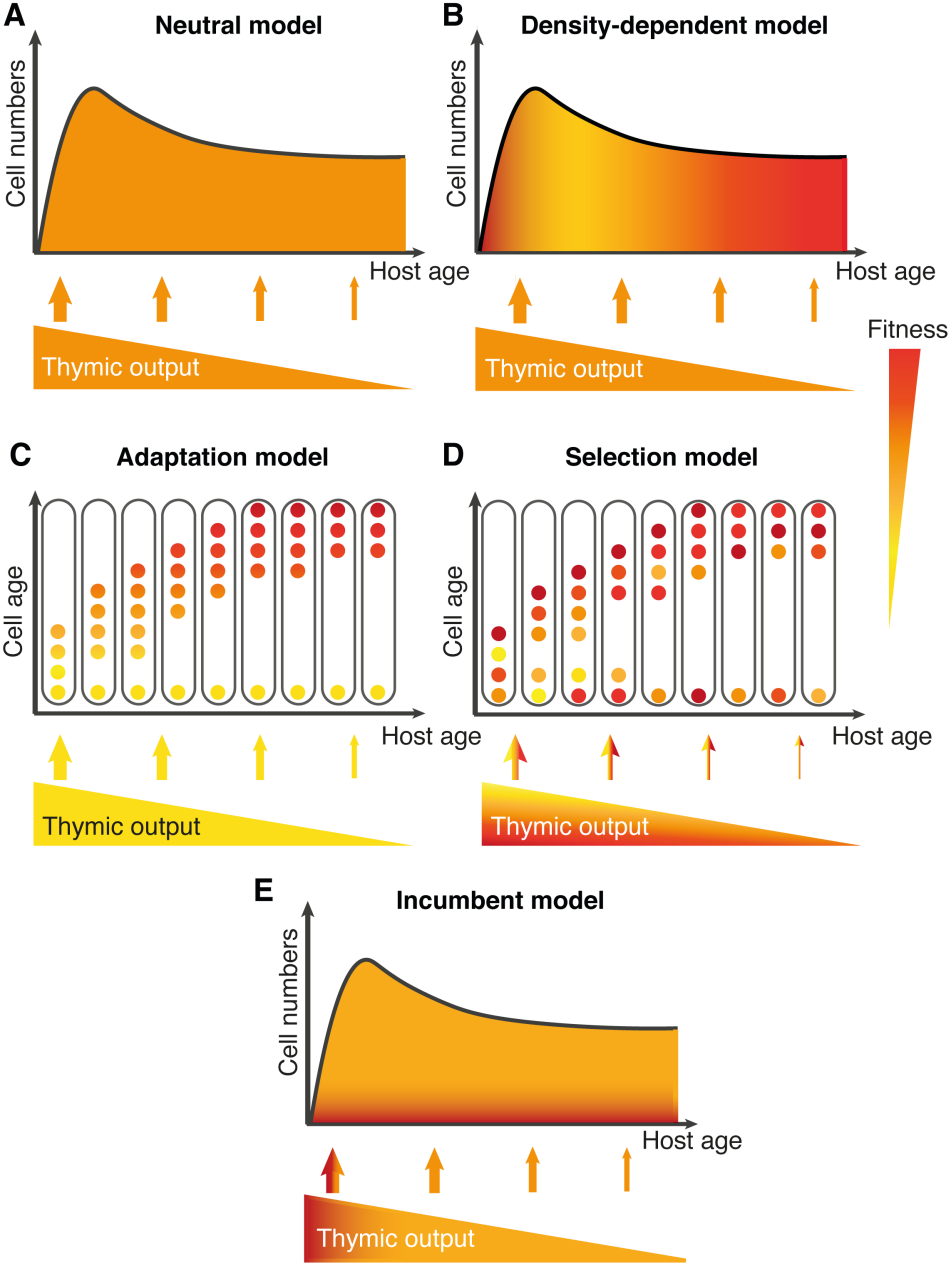
Models of naive T-cell homeostasis. (A) A simple random birth–death model, with both processes occuring at rates that are constant over time and identical for all cells. (B) A model of density-dependent homeostasis in which every cell’s homeostatic fitness declines equally with total population size, due to resource competition or other forms of quorum-sensing. (C) A model of adaptation in which every cell’s fitness increases progressively with its post-thymic age. We assume that post-thymic age is inherited by a dividing cell’s offspring. (D) A model in which T cells are generated with a distribution of intrinsic, constant, and heritable fitnesses. Over time, selection acts on this distribution. (E) A variant of the selection model, in which the naive pool comprises numerically stable, self-renewing ‘incumbent’ cells generated early in life and ‘displaceable’ cells that are continually replaced by new emigrants from the thymus.

We explored five types of behaviour underlying naive T-cell population dynamics, which we describe conceptually and mathematically here and illustrate in Fig 1. Full details of the formulation and solution of these models as well as the procedures for fitting them to experimental data are given in S1 Text. We use the standard definition of cell ‘fitness’ as a measure of reproductive success, or a net growth rate—i.e., the propensity of a cell for division minus its propensity for loss. Therefore, ‘homeostatic fitness’ is an absolute, not a relative, measure of the ability of a T cell and its progeny to persist within the naive T-cell compartment over time.

#### Neutral

Here, all naive cells are assumed to have equal fitness and obey a simple random birth–death process, in which mature naive cells are released from the thymus at a time-dependent rate *θ*(*t*) and form a kinetically homogeneous population *N*(*t*) that self-renews through division and is lost due to death or differentiation (Fig 1A). The net, constant per capita rate of loss in the periphery is *λ* (the negative of cell fitness);

$$\frac{dN(t)}{dt}=\theta (t)-\lambda N(t).$$ (1)

#### Density-dependent

Cells are again assumed to be kinetically homogeneous, but rates of division and/or loss vary with total population size *N*(*t*) through a quorum-sensing mechanism such as competition for a shared, limiting resource (Fig 1B);

$$\frac{dN(t)}{dt}=\theta (t)-\lambda (N)N(t).$$ (2)

#### Adaptation

Here, the fitness of cells and their offspring increases with time spent in the periphery. We refer to this as the post-thymic age, *a*. This ‘age conditioning’ may be a programmed, cell-intrinsic effect or the result of repeated interactions of cells with their environment. Increasing fitness with post-thymic age will lead to a preferential accumulation of older cells with time, whether or not there is any competition for resources (Fig 1C). In this model, the homeostatic dynamics are heterogeneous with respect to post-thymic age *a*, and the expected population density *N*(*a*,*t*) evolves according to the following partial differential equation

$$\frac{\partial N(a,t)}{\partial a}+\frac{\partial N(a,t)}{\partial t}=-\lambda (a)N(a,t),$$ (3)

where *λ*(*a*) is the net rate of loss, assumed to be a decreasing function of post-thymic age. The rate of thymic export is the rate of production of cells of age zero, *N*(0,*t*) = *θ*(*t*). The total population size in a host of age *t* is N(t)=∫N(a,t)da.

#### Selection

An alternative model of heterogeneity is one of selection acting on natural variation in fitness within the population. Such variation might derive from diferences in TCR affinity for self-peptide–MHC ligands or heritable differences in expression of receptors for homeostatic stimuli [21–23, 25]. We assume that the fitness of each cell leaving the thymus is drawn from a lognormal distribution *f*_*θ*_(*λ*) and is passed on to both daughter cells following division.

$$\frac{dN(\lambda ,t)}{dt}=\theta (t)f_{\theta }(\lambda )-\lambda N(\lambda ,t).$$ (4)

In this scenario, cells with higher fitness (lower net loss rate *λ*) accumulate over time (Fig 1D).

#### Incumbent

We also consider a variant of a selection model, described in Hogan et al. [13], in which the naive pool comprises 2 subpopulations in distinct homeostatic niches—a small, stable population (‘incumbent’ cells) established within 8 wk of birth as well as a larger pool of ‘displaceable’ cells that can be replaced by thymic emigrants (Fig 1E). We refer to this as the incumbent model.

### Busulfan chimeras

The protocol used to generate busulfan chimeras is described in detail elsewhere [13, 26].

### Flow cytometry

The following monoclonal antibodies and cell dyes were used: CD45.1 FITC, CD45.2 AlexaFluor700, CD45.2 FITC, TCR-beta APC, CD4 PerCP-eFluor710, CD44 APC-eFluor780, CD25 PE-Cy7, L-selectin eFluor450, CD122 biotin (all eBioscience); CD8 Pacific Orange, streptavidin PE-TexasRed, LIVE/DEAD blue (all Invitrogen); and CD45.1 Brilliant Violet 650, CD4 Brilliant Violet 711, and TCR-beta PerCP-Cy5.5 (all BioLegend). Samples were acquired on LSR-II, LSRFortessa, or Fortessa X20 flow cytometers (BD), and analysis was performed with FlowJo software (Treestar).

### Ethics statement

Wild-type (WT) CD45.1 and CD45.2 mice were bred and maintained in conventional pathogen-free colonies at either the National Institute for Medical Research (London, United Kingdom) or at the Royal Free Campus of University College London. All experiments were performed in accordance with UK Home Office regulations, project license number PPL70-8310.

## Results

### Naive T-cell population dynamics in healthy mice and post-thymectomy

The first test of the models was to confront them with data from a study that measured the numbers of naive (CD62L^hi^CD44^lo^) CD4 and CD8 T lymphocytes recovered from spleen and lymph nodes in euthymic (WT) and thymectomised (Tx) C57BL/6 mice, from soon after birth up to 65 wk of age [8]. Thymectomy was performed at 7 wk of age.

**Fig 2:**
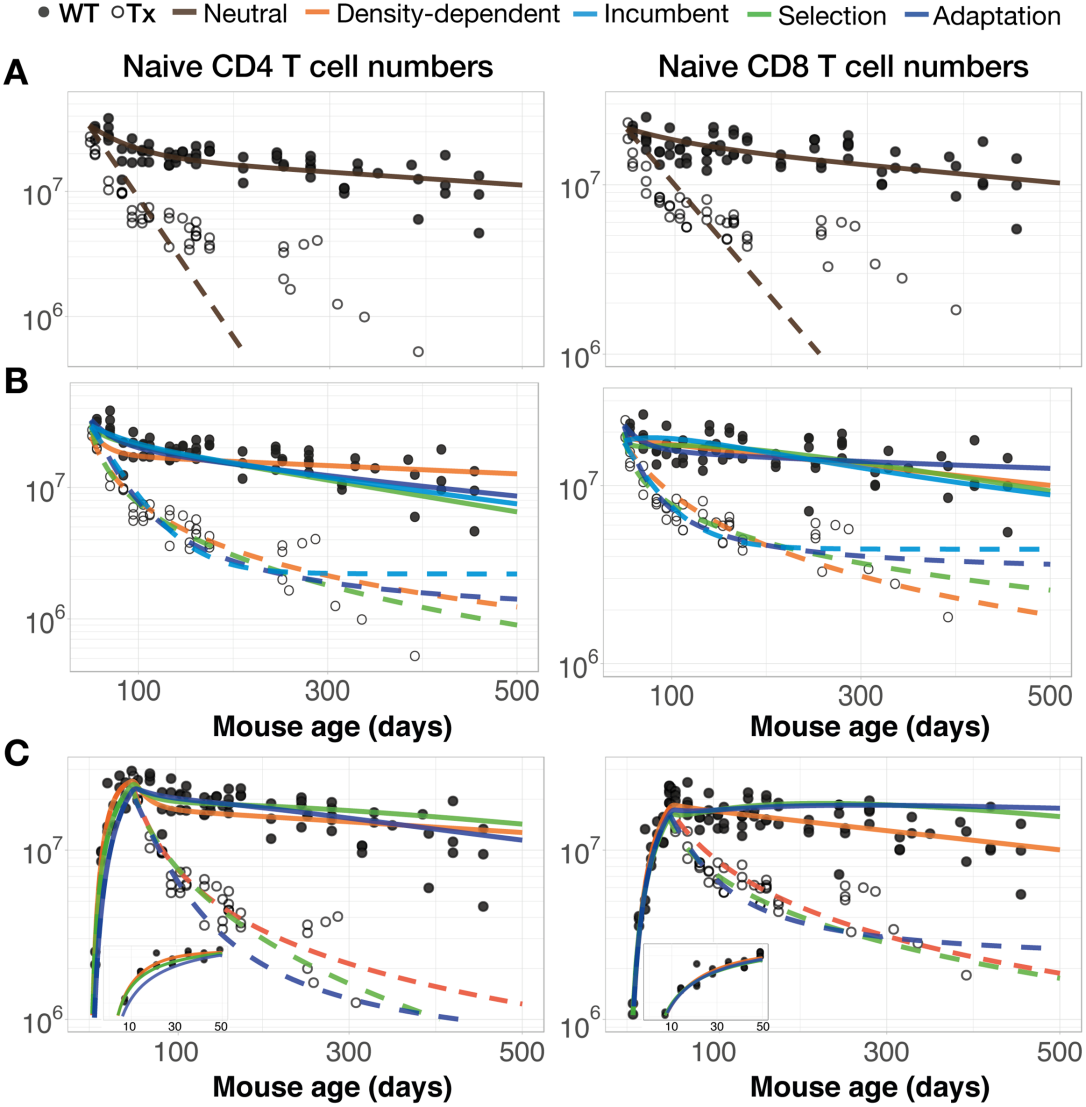
Comparison of the ability of models to explain naive T-cell dynamics in euthymic and Tx mice. (A) The neutral model describes replete kinetics well but fails to capture the slow loss of cells following thymectomy. (B) Comparing the density-dependent, adaptation, selection, and incumbent models using data from thymectomy onwards. (C) The same comparisons but using data from birth. Insets detail the growth of naive T-cell numbers in young mice. Data are provided in S1 Data. Tx, thymectomised; WT, wild-type.

We found that the neutral model of random division and loss at constant rates throughout life reproduces the long-term kinetics of both naive CD4 and CD8 T cells in WT mice remarkably well (Fig 2A, solid lines). These changes in naive T-cell numbers in mice under normal conditions can therefore be explained broadly without invoking any compensatory changes in the division or cell longevity with pool size or waning thymic output. However, the neutral model clearly fails to capture the kinetics with which naive CD4 and CD8 T-cell numbers fall following thymectomy (Fig 2A, dashed lines). In the original study of den Braber et al. [8], a resource competition model was invoked to explain the improved net survival of cells in the absence of thymic export. We replicated their fits using the same model, confirming that a density-dependent net rate of loss provides a substantially better description of both naive CD4 and CD8 kinetics—in WT and Tx mice simultaneously from 7 wk of age—than the neutral model (Fig 2B, orange lines; ΔAIC = 91 and 115, respectively; ΔAIC refers to differences in the Akaike Information Criterion [27]).

Given the evidence for heterogeneity within the naive compartments, we then assessed the abilities of the adaptation, selection, and incumbent models to explain naive T-cell kinetics from the age at thymectomy (7 wk) onwards, comparing them to the density-dependent model. The adaptation, selection, and density-dependent models produced visually similar fits (Fig 2B), but the selection model received the strongest statistical support (Table 1, ΔAIC = 12). In contrast, the incumbent model gave the poorest fit (ΔAIC = 29). Therefore, naive CD4 and CD8 T-cell kinetics from 7 wk of age onwards in euthymic and Tx mice—during which cell numbers vary by a factor of approximately 10—can be explained well without invoking any density-dependent processes.

**Table 1:** Comparison of AIC values for different models fitted to published observations of naive T-cell counts in normal and Tx mice [8] and to data from busulfan chimeric mice. For model comparison, only differences in AIC, not absolute values, are meaningful—these differences are shown relative to the best-fitting model, with larger numbers indicating lower support.

Population	Dataset	Model and ΔAIC			
		Selection	Adaptation	Density-dependent	Incumbent
Naive CD4	WT and Tx data from thymectomy (ref. [8])	0	16	12*	29
	WT and Tx data from birth (ref. [8])	3.6	9.2	0	—^†^
	Busulfan chimeras	24	9.5	390*	0
Naive CD8	WT and Tx data from thymectomy (ref. [8])	0	11	27*	23
	WT and Tx data from birth (ref. [8])	66	71	0	—^†^
	Busulfan chimeras	27	4.0	440*	0

*Initial values of cell numbers for the density-dependent model were determined from the counts observed at 7 wk in euthymic mice [8], or from age at the earliest bone marrow transplant for the busulfan chimeras.

^†^The incumbent model cannot be fitted to the counts from birth because the developmental dynamics of any incumbent naive subpopulations in neonates are unknown.

Abbreviations: AIC, Akaike Information Criterion; Tx, thymectomised; WT, wild-type.

The data included observations from mice soon after birth, when mice might be considered lymphopenic [28], and so any density-dependent effects might be more apparent. Assuming that thymocyte numbers from birth into old age continue to reflect thymic output with the same constant of proportionality, we fitted the density-dependent, adaptation, and selection models to the entire time courses (Fig 2C). It was not possible to fit the incumbent model to these data because the kinetic by which any such cells are established is unknown. Here, we found greater support for the density-dependent model over the selection and adaptation models (ΔAIC = 3.6 and 9.2, respectively, for CD4; 66 and 71, respectively, for CD8), presumably due to its improved description of the additional data describing the growth of naive T-cell numbers in young mice. To examine this more closely, we fitted the neutral and density-dependent models to the data up to 7 wk only, a period during which naive T-cell numbers increase rapidly. In this early window, we found that the neutral and density-dependent models had essentially equivalent statistical support (ΔAIC = 0.43 in favour of the neutral model for CD4; 0.02 for CD8). Therefore, our analysis provides no strong evidence that lymphopenia-induced proliferation through reduced competition for homeostatic stimuli is a dominant factor in establishing the naive CD4 and CD8 T-cell pools early in life.

### Modeling the kinetics of naive T-cell replacement in mice

**Fig 3:**
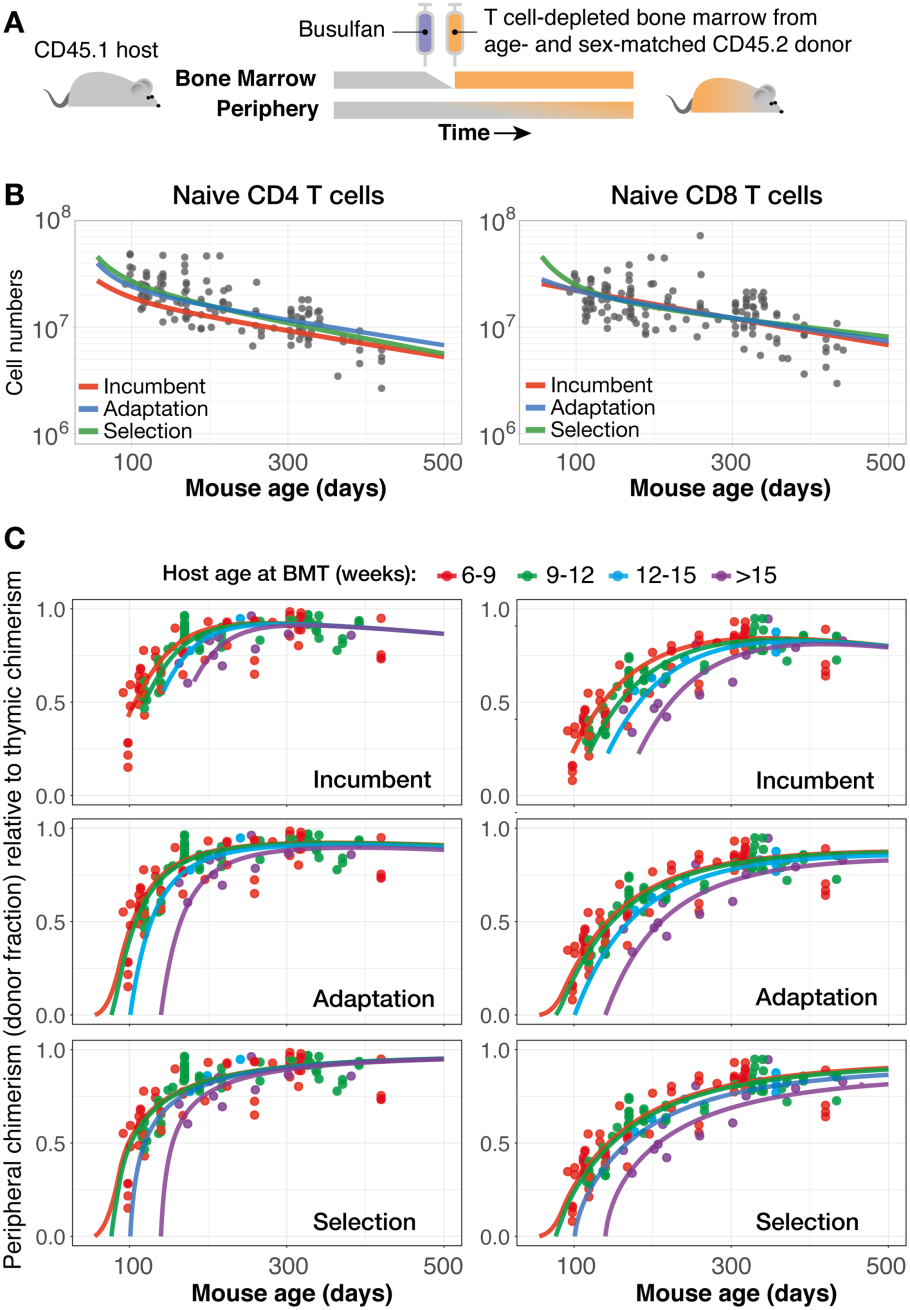
Comparison of models explaining naive T-cell dynamics in busulfan chimeras. (A) Generating busulfan chimeras. (B) Fitting the incumbent, adaptation, and selection models to naive CD4 and CD8 counts from busulfan chimeras made in recipients of different ages. (C) Simultaneous fits to the normalised chimerism in the peripheral naive CD4 and CD8 pools. Colours indicate different age groups of recipient mice. The predictions shown were generated using the mode of the age within each group. Data are provided in S2 Data. BMT, bone marrow transplant.

Previously, we used a bone marrow chimeric system to study the homeostatic dynamics of T cells [13, 29]. CD45.1 C57BL/6 mice were treated with optimised doses of the transplant-conditioning drug busulfan, specifically ablating haematopoetic stem cells (HSCs) but leaving thymic and peripheral lymphocyte compartments intact. Following congenic (CD45.2) bone marrow transplant (BMT), the appearance of donor-derived cells serves as a proxy for quantifying bone marrow output following BMT (Fig 3A). Therefore, the combination of the kinetics of the compartment sizes and the infiltration of donor-derived cells (Fig 3B and 3C) allows us to quantify rates of turnover and the rules of replacement. The replacement of naive (CD62L^hi^ CD44^lo^ CD25−) CD4 and CD8 T-cell compartments post-BMT stabilise at 80% to 90% of the levels in the thymus [13]. This shortfall is not an artefact of the experimental manipulation because both *γδ* T-cell and B cell populations in the same mice undergo near 100% replacement post-BMT (Hogan, Rane, Yates, and Seddon, unpublished observations). The incomplete replacement of host naive cells could not be explained by thymic involution nor by any homogeneous models in which all cells obey the same kinetics [13] and which include the neutral and density-dependent models considered here. Rather, we found support for a model in which slowly self-renewing, ‘incumbent’ subpopulations of naive CD4 and CD8 T cells established early in life resist displacement by naive T cells generated later in life. In that study, we found marginally lower support for an adaptation model identical to the one described here. However, because the observations came from mice that underwent BMT at identical ages, our ability to dissociate any effects of host age and cell age was relatively limited.

To address this problem, we performed additional experiments analysing the reconstitution dynamics of busulfan chimeras in hosts whose age at BMT ranged from 6 wk to 6 mo (Materials and methods; data are provided in S2 Data). This expanded range provided a richer dataset, requiring models to be able to account for any impact of the ageing host environment as well as of cell age. Models were fitted simultaneously to the time courses of total naive T-cell numbers and the normalised peripheral chimerism (i.e., the donor proportion in the peripheral naive T-cell population divided by the stable donor proportion in all thymic compartments). First, as a consistency check, we fitted a density-dependent model and found that it described the data poorly (Table 1 and S1 Fig), supporting our original conclusion that homogeneous models are unable to explain the replacement kinetics in this system. In contrast, the incumbent, adaptation, and selection models yielded good and visually similar fits to both total cell numbers (Fig 3B) and peripheral chimerism (Fig 3C) for both naive CD4 and CD8 T cells. Within this group of candidates, the incumbent model had the strongest support, and the selection model had the weakest (Table 1). However, recall that the incumbent model described the long-term kinetics of T-cell numbers in WT mice and mice thymectomised at 7 wk of age relatively poorly (Table 1). Therefore, by assessing both quality of fit and model robustness, we find (i) relatively little support for the incumbent model and (ii) equivocal support for the adaptation and selection models, which—to opposing degrees—are able to explain both the WT/Tx data and the replacement kinetics in the busulfan chimeras.

### Predicting the loss of young and old naive T cells in young recipient mice

**Fig 4:**
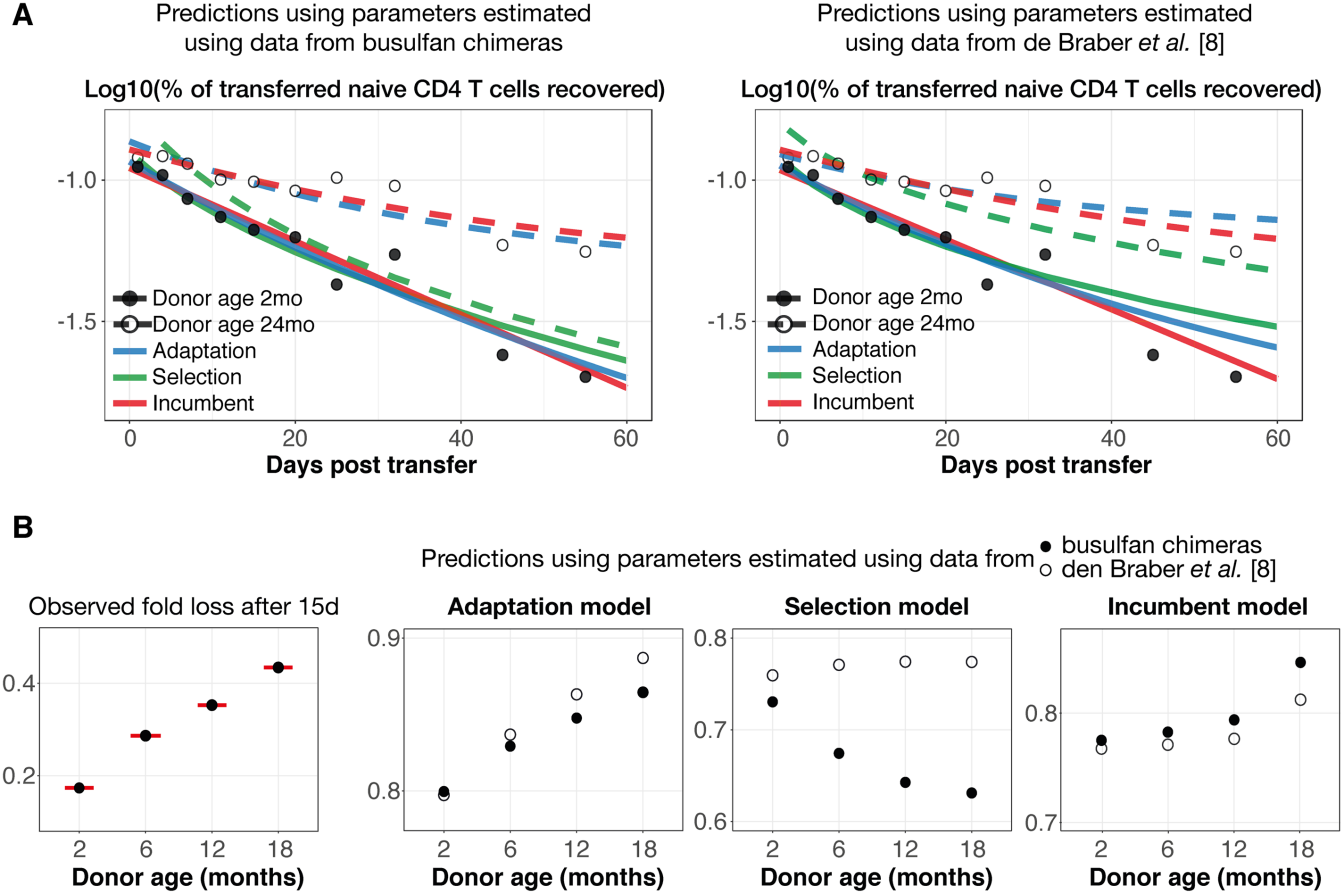
Discriminating between models of heterogeneous turnover by simulating the adoptive transfer experiments reported in ref. [14]. (A) Observed and predicted kinetics of loss of naive CD4 cells taken from young (2 mo) (solid circles and solid lines) and aged (24 mo) (open circles and dashed lines) donors, using the adaptation (blue line), incumbent (red lines), and selection (green lines) models. Data were taken from Fig 2C in ref. [14]. Parameters were taken from the fits to the data from WT and Tx mice reported in ref. [8] and from our busulfan chimera data (left and right panels, respectively). (B) Predicting the trend in the loss of donor cells from the naive CD4 pool over 15 d, using AND TCR transgenic naive T cells taken from donors of four different ages (left panel, data reproduced from Fig. 4E in ref. [14]). The three panels to the right show the models’ predicted fold loss of polyclonal naive CD4 and CD8 T cells 15 d after adoptive transfer, from donors of the same ages used in the AND adoptive transfer experiment. Due to intrinsic differences in the survival rates of AND and polyclonal naive cells, the absolute levels of recovery differ substantially, but the predicted trends show that only the adaptation model is able to reproduce a consistent increase in recovery with donor age. As in panel A, these predictions were generated using model parameters obtained from fits to the data from the busulfan chimeras (solid circles) and from ref. [8] (open circles). Data are provided in S3 Data. TCR, T-cell receptor; Tx, thymectomised; WT, wild-type.

The adaptation, selection, and incumbent models all exhibit differential growth or loss of populations due to population-level variability in homeostatic fitness. This variability may derive from progressive conditioning with time spent in the periphery (adaptation), be generated during thymic development (selection), or be rooted in differences in fitness between the cells that populate the empty neonatal enivironment and those that enter the replete naive compartment later in life (the incumbent model). In all three scenarios, more proliferative or longer-lived naive T cells accumulate, but the mechanisms are difficult to directly distinguish experimentally without tracking the fate and fitness of individual cells over long timescales. Aiming to differentiate the mechanisms further, we turned to data from a study in which naive CD4 T cells from donor C57BL/6 mice of different ages were transferred into young recipients (ref. [14] and S3 Data). In that study, polyclonal naive CD4 T cells from older hosts clearly exhibited a survival advantage over those from younger hosts over the 20 d following adoptive transfer (Fig 4A). To test the abilities of the adaptation, selection, and incumbent models to explain these data, we simulated the experiment using these models to predict the kinetics of cohorts of naive CD4 T cells sampled from hosts aged 2 and 20 mo (Fig 4A). We performed these simulations with two sets of parameters—those estimated using the data in den Braber et al. [8] and those from the busulfan chimeras. To compare these predictions with the data, we estimated only a single quantity: the number of cells recovered immediately following the transfer. The selection model failed to reproduce the kinetics of donor cell loss. In contrast, the adaptation and incumbent models predicted these kinetics remarkably accurately for both sets of parameters (Fig 4A), with adaptation receiving the strongest statistical support (Table 2).

**Table 2:** Comparison of AIC values for models fitted to published observations of proportions of cells transferred in WT mice from donors with different ages (2 and 24 mo), to predict the kinetics of donor cell loss [14].

Data	Model and ΔAIC		
	Adaptation	Selection	Incumbent
WT and Tx data (ref. [8])	0	13	3.8
Busulfan chimeras	0	25	1.7

Abbreviations: AIC, Akaike Information Criterion; Tx, thymectomised; WT, wild-type.

In another experiment reported in Tsukamoto et al. [14], TCR transgenic (AND) naive CD4 T cells from C57BL/10 donors of ages 2, 6, 12, and 18 mo were transferred into young hosts, revealing a progressive increase in cell survival with donor age. A key observation was that the loss rate of cells derived from 12-mo-old donors was significantly greater than of cells from 18-mo-old donors. The authors argued that because thymic output drops to low levels by 6 mo of age in mice [2, 30], any selection for fitter cells ought to be complete by 1 year of age, and so there should be little difference in the rates of loss of cells from donors older than this. They therefore favoured adaptation over selection as an explanation for these data. To examine this argument quantitatively, we simulated the experiment using the adaptation, incumbent, and selection models, in each case using the parameters from fits to both the data from den Braber et al. (Fig 2) and our busulfan chimeras (Fig 3). Notably, Tsukamoto et al. consistently observed that the AND transgenic T cells were lost more rapidly than polyclonal T cells from age-matched mice, and so our model predictions—derived from polyclonal T-cell dynamics—overestimated the observed rates of survival of AND T cells over 15 d post-transfer (reproduced in Fig 4B, left panel). However, all three models allowed us to predict the trend in 15-d survival with donor age (Fig 4B). We confirmed the argument in Tsukamoto et al. [14] that adaptation, but not selection, can generate a consistent increase in the recovery of cells from donors up to 18 mo old. The incumbent model also fails to capture this trend, predicting a similar fold loss for 2-, 6-, and 12- mo-old donors. This is because the proportion of the pool occupied by the more persistent incumbent cells is predicted to increase only slowly with host age [13].

We conclude that adaptation is the strongest candidate model of naive T-cell homeostasis that we considered, in terms of both the quality of its descriptions of the data and the diversity of datasets it could describe (Table 3).

**Table 3:** Comparing the explanatory power of models of naive T-cell homeostasis in mice. Here, we disregard statistical distinctions between models and simply assess their ability to describe the key features of experimental datasets. A 'X' indicates that the model is obviously inadequate in that setting.

Data	Homogeneity		Heterogeneity		
	Neutral	Density-dependent	Incumbent	Selection	Adaptation
Cell counts in WT mice	✓	✓	✓	✓	✓
Cell counts in Tx mice	X	✓	X	✓	✓
Donor fractions in Busulfan chimeras	—	X	✓	✓	✓
Kinetics of transferred cells	—	—	X	X	✓

Abbreviations: Tx, thymectomised; WT, wild-type.

### Estimates of biological quantities generated by the adaptation model are largely consistent between different datasets

**Fig 5:**
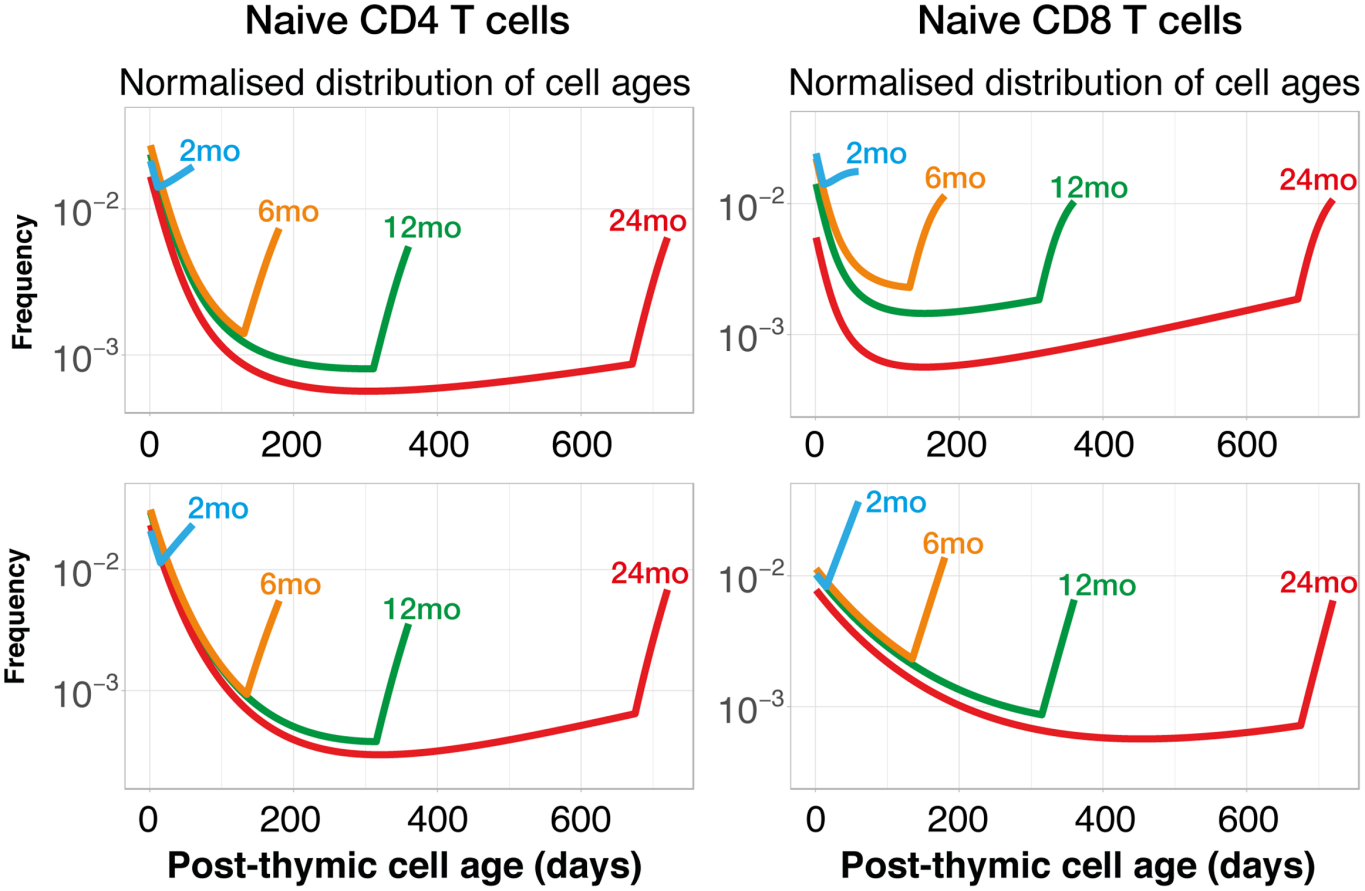
Predicted post-thymic cell age distributions, for different host ages, using the adaptation model. The normalised age distributions of naive CD4 and CD8 T cells were generated using parameters obtained from fits to the data from den Braber et al. [8] (upper panels) and from the busulfan chimeras (lower panels). Different coloured curves denote different host ages. Distributions illustrate the preferential accumulation with longer-lived and/or more proliferative cell populations with host age. The discontinuity in the gradients at older cell ages derives from uncertainty in the precise form of the age distribution of cells at the beginning of the experiment. We proposed various forms for this distribution, and the parameters of each were estimated from the data. However, for all distributions we explored, the same U-shaped trend emerges over time.

Another test of a model is the extent to which its parameters agree across fits to multiple datasets. We therefore compared the parameters that we derived from the data from den Braber et al. [8] and the busulfan chimeras. We found consistent estimates of the naive CD4 or CD8 T-cell pool sizes and rates of thymic output at 7 wk of age (Table 4), but estimated that fitness increases more rapidly with cell age in the WT/Tx mice studied in ref. [8] than in the busulfan chimeras. This difference is more evident for naive CD8 T cells, largely because den Braber et al. saw a rapid loss of these cells immediately post thymectomy, followed by a slower decline (Fig 2, right hand column), consistent with a more bimodal distribution of cell lifetimes with cell age than the smoother, exponential one we inferred from the busulfan chimera data. Correspondingly, the two datasets were best described with different functional forms of the declining loss rate *λ*(*a*). Nevertheless, the two models give very similar predictions of the age structure of the naive CD4 T-cell pool for different host ages (Fig 5). Both show a high preponderance of younger cells—consistent with the consensus that there is a dominant role for thymic export in maintaining naive T-cell numbers in mice [8, 13]—and a clear accumulation of older cells over time.

**Table 4:** Comparison of parameter estimates obtained from different datasets. 95% CIs are shown in parentheses. See S1 Text for a description of the models.

Population	Parameter		Data	
			WT and Tx data (ref. [8])	Busulfan chimeras
Naive CD4	Cell numbers at 7 wk	*N*_0_ × 10^−6^	33 (30, 40)	50 (42, 86)
	Daily thymic output at 7 wk	*θ*_0_ × 10^−6^	0.63 (0.36, 1.03)	0.85 (0.73, 1.1)
	Net loss rate of cells of age 0 (d^−1^)	*λ*_0_	0.044 (0.022, 0.12)	0.050 (0.039, 0.060)
	Timescale of increase in fitness (d)	*r*	68 (30, 110)	120 (100, 140)
Naive CD8	Cell numbers at 7 wk	*N*_0_ × 10^−6^	22 (19, 24)	30 (22, 42)
	Daily thymic output at 7 wk	*θ*_0_ × 10^−6^	0.50 (0.34, 0.60)	0.29 (0.26, 0.34)
	Net loss rate of cells of age 0 (d^−1^)	*λ*_0_	0.063 (0.026, 0.097)	0.019 (0.015, 0.025)
	Timescale of increase in fitness (d)	*r*	33 (23, 73)	250 (190, 370)

Abbreviations: Tx, thymectomised; WT, wild-type.

### Does maturation of RTEs underlie the process of adaptation in homeostatic fitness?

Newly developed T cells (RTEs) take time to reach full functional competence in the periphery [16, 17], and it seems plausible that this transition may be accompanied by changes in their capacity to survive or self-renew. It is therefore possible that the adaptation process we model, in which homeostatic fitness changes with post-thymic age, may reflect the process of RTE maturation.

In our analyses of the adaptation model, we explored net loss rates *λ*(*a*) that decline smoothly with cell age, either exponentially or with a sigmoid form (S1 Text). While RTEs remain difficult to define precisely and thus the kinetics of their maturation remain unclear, we explored an alternative model of adaptation in which RTEs mature abruptly after a fixed time *a*_M_ in the periphery and have a distinct loss rate (*λ*_RTE_) from that of mature naive cells (*λ*_M_). As expected, we found that this stepwise, or ‘conveyor belt’, RTE maturation model predicted that mature naive cells are lost at a lower net rate than RTEs. However, the model yielded inferior fits to naive CD4 and CD8 counts in WT and Tx mice (ΔAIC = 83 for CD4 and 64 for CD8 cells) and fitted the data from the busulfan chimeras poorly (not shown). Nevertheless, the model predicted maturation times *a*_M_ of 6 and 2 wk for CD4 and CD8 T cells, respectively, which are in approximate agreement with the estimate of 3 wk for CD4 and CD8 RTEs combined in the studies by Berzins et al. [31, 32] and closely comparable to a more recent estimate of the expected time to maturation of CD4 RTEs [33]. We conclude that there is little support for a deterministic, conveyor belt model in which maturation of RTEs is accompanied by a rapid increase in homeostatic fitness. However, because the true kinetics of RTE maturation are unknown and may be more extended in duration, we cannot rule it out as the biological underpinning of any more gradual increase in fitness with cell age.

### Recovery of naive T-cell numbers following depletion—Does the naive T-cell pool have a memory?

**Fig 6:**
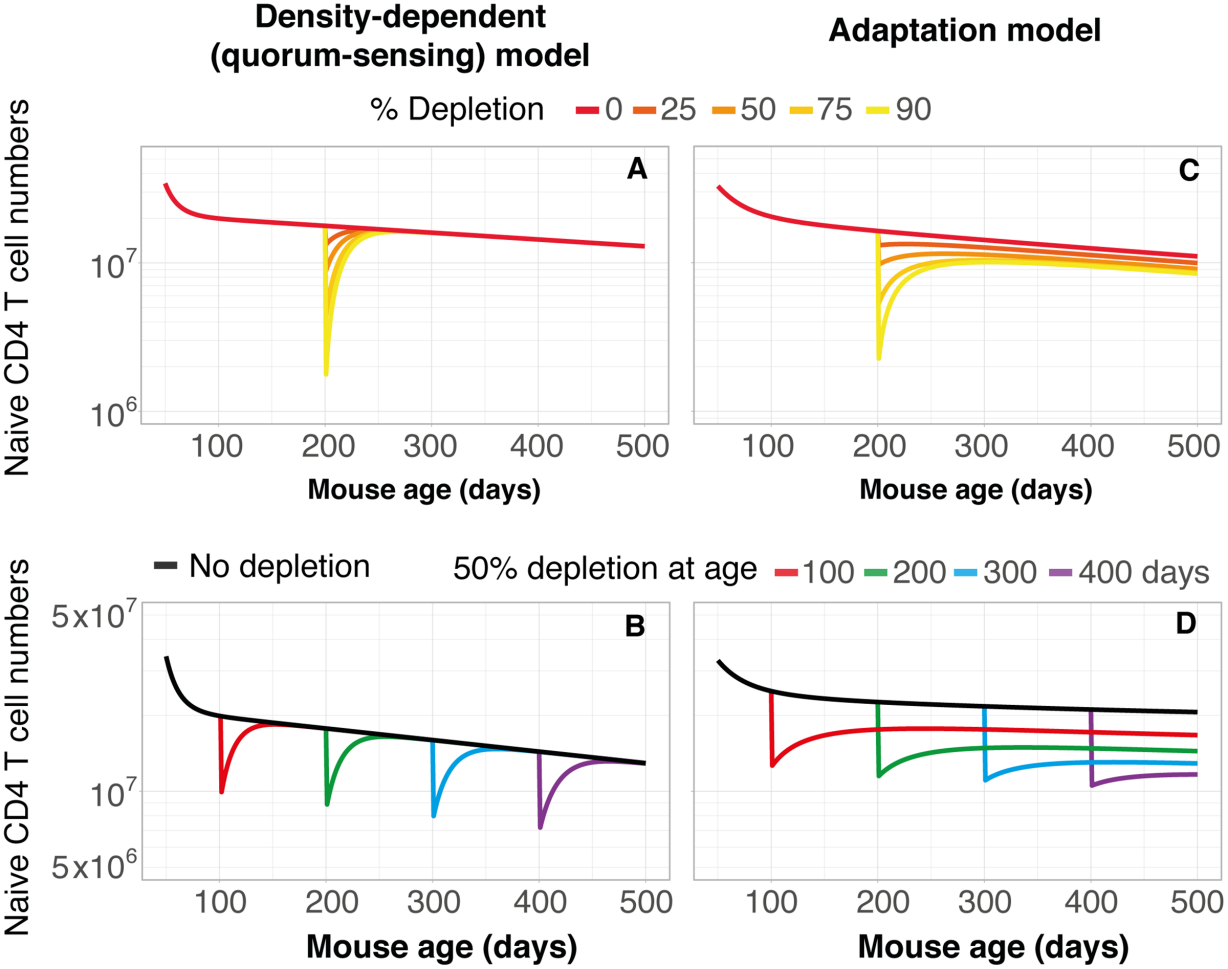
Simulations of recovery of naive CD4 T-cell numbers following depletion. Under the assumptions of pure density-dependent regulation of naive T-cell numbers (left panels), or the adaptation model (right panels), we simulated recovery from different levels of naive CD4 T-cell depletion at age 200 d (upper panels) and recovery from 50% depletion at different host ages (lower panels). Simulations were performed using the model parameters estimated from the data in ref. [8].

Quorum-sensing models such as the one expressed in Eq 2 predict a unique set point or carrying capacity for the naive T-cell pool size for any given level of thymic output. In such models, following transient depletion, T-cell numbers will eventually rebound to those in healthy age-matched animals, irrespective of the extent of depletion (Fig 6A) or the age of the animal (Fig 6B). This recovery happens on a timescale dictated by the rate of turnover. Here, the naive pool is essentially ‘memoryless’, in the sense that there is no imprint of the pool’s developmental history on its potential for recovery. In contrast, in a purely adaptive model of homeostasis, there is no compensatory proliferative renewal of existing cells under lymphopenia, and cells behave essentially independently and according to their post-thymic age. In this regime, there is no unique set point for naive T-cell numbers. The extent of recovery is dictated by the extent of depletion (Fig 6C) and is further limited in older mice due to waning thymic output (Fig 6D). We would expect the latter effect to be even stronger in humans, who experience a more substantial drop in thymic output with age [8, 34–36]. Indeed, the correlation between loss and recovery predicted by the adaptation model (Fig 6C) is strikingly similar to the observation that pretreatment numbers of naive CD4 T cells in HIV-infected patients are a strong predictor of the recovery of the CD4 T-cell pool following antiretroviral therapy [37–40]. The predictions in Fig 6D also echo the observation that the potential for restoration of naive T-cell numbers in HIV-infected patients is progressively impaired with age [38, 41, 42]. While compensatory proliferation does occur in severely T cell–depleted humans, we speculate that the history of the T-cell pool, reflected in its age structure, may also be a determinant of its capacity for reconstitution.

## Discussion

Discussions of naive T-cell dynamics often focus on the ability of these cells to sense ‘space’ and that competition between and within TCR clones for resources determines the pool size and shapes its diversity [7, 43–46]. However, it is clear that there is also heterogeneity in the homeostatic fitness of individual T cells that may relate to their developmental status in the periphery, intrinsic variation programmed during ontogeny, or to changes induced by interactions with their microenvironment over time. Given this heterogeneity, we questioned here whether quorum-sensing is indeed the dominant regulatory mechanism under normal physiological conditions and asked whether any one of these homeostatic forces can best account for multiple readouts relating to the long-term dynamics of the naive pool. Remarkably, a model of continuous adaptation, in which homeostatic fitness increases with cell age, emerged as the only mechanism capable of explaining all of the datasets (Table 2). While its visual descriptions of the data were consistently good, the model of adaptation was not statistically favoured as the best model in all cases. Nevertheless, the universality of its performance across multiple independent datasets weighs strongly in the model’s favour, particularly because alternatives that are strong competitors in one setting clearly fail to explain observations in another.

Studying the kinetics of donor fractions in busulfan chimeric mice that are thymectomised at different times post BMT may provide additional empirical evidence to help distinguish the various models tested in this study (S2 Fig, panel A) because it would allow one to track population age structures at different levels of lymphopenia. We simulated such an experiment using parameters estimated from the fits to busulfan chimera data and found that the adaptation, selection, and incumbent models make distinct predictions regarding the dynamics of chimerism within the naive pools (S2 Fig, panel B).

While the model of adaptation in isolation proved most robust when fitted to diverse datasets, we do not exclude it acting in combination with other homeostatic mechanisms. Selection for fitter cells alone fails to explain all the data, but given the natural variation in TCR affinity for self-peptide–MHC, we might expect some level of accumulation of cells at the upper end of acceptable self-reactivity with age. Furthermore, the data we analysed in this study related to the natural history of naive T-cell numbers across the full lifespan of healthy mice and the partially depleted compartments induced by thymectomy in young adulthood. Quorum-sensing did not need to be invoked over this wide range of cell numbers, but there is evidence for it in more profoundly lymphopenic conditions [11, 13, 47–51], and so it likely acts in concert with adaptation under these conditions.

T-cell receptor excision circles (TRECs) are nonreplicating circular fragments of DNA that result from TCR rearrangement in the thymus and are distributed at random to daughter cells during mitosis. Their frequency in a naive T-cell population is determined by their rate of influx from the thymus and the diluting effects of cell division. TRECs therefore are potentially informative regarding homeostatic mechanisms. den Braber et al. [8] found (i) no significant decline in TREC frequencies within splenic naive CD4 and CD8 T cells with mouse age and (ii) a drop in TREC content following thymectomy in naive CD8 T cells (spleen) and both naive CD4 and CD8 T cells (lymph nodes). These observations can be reconciled if one takes into account the constant replenishment of TREC-rich naive T cells from the thymus in healthy mice, which exceeds the rate of cell production by peripheral division. In healthy mice, any reduction of TREC frequencies through division, or by the preferential accumulation of relatively TREC-depleted older cells through increased survival, will then be countered by the influx of RTEs, which remains considerable across a mouse lifetime. The first observation therefore gives us little information with which to distinguish competition and adaptation. Similarly, TREC decline in Tx mice can be explained by either mechanism. With adaptation, the combination of slow peripheral division but preferential survival of older, TREC-depleted, naive T cells drives down the average TREC content when the TREC-rich RTE supply is removed.

On similar lines, Thomas-Vaslin et al. [49] studied an experimental system in which apoptosis was chemically induced in dividing cells both in the thymus and the periphery. In otherwise healthy mice, they found that the subsequent approximately 50% drop in naive T-cell numbers was equivalent to that incurred by thymectomy, but the same treatment in Tx mice had no impact on naive T-cell numbers over a 2-wk period. These observations are consistent with the consensus that the thymus is the dominant source of naive T-cell production in mice and that there is little or no increase in peripheral division to compensate for thymectomy [52]. Thomas-Vaslin et al. found that in healthy mice, naive T-cell numbers returned to normal levels within 10 wk of treatment. We found that with an active thymus, numbers can be restored to close to healthy levels in a model of adaptation within a similar time frame (Fig 6C). The combination of these observations lends further weight to the explanatory power of the adaptation model under replete or partially lymphopenic conditions.

Our models do not address the underlying mechanism driving adaptation, though it seems likely to be due to the accumulation of signals from the cells’ environment, similar to other biological systems in which recurrent signals lead to cellular adaptation. T cells might also modify their sensitivity to homeostatic cues in response to regular interactions with self-peptide–MHC ligands and cytokines such as IL-7 [53–55]. Whatever the causal mechanism, survival may be the key property that is subject to adaptation in mice. Progressive alterations in intracellular prosurvival factors such as Bim and Erk kinases have been implicated in increasing the longevity of naive T cells [14, 56], while levels of homeostatic proliferation in mice do not appear to increase with age [13]. Overall, then, we argue from parsimony that the main determinants of naive T-cell numbers in healthy mice are (i) thymic output, which is very significant in early life but declines with age; (ii) low and relatively constant levels of renewal by homeostatic proliferation; and (iii) a gradual increase in cell longevity with post-thymic age. This is an especially important conclusion in relation to comparisons of adaptation and selection; these are fundamentally distinct processes, but both result in an increase of average cell fitness with time and are difficult to distinguish directly with experiments alone. In humans, proliferation of naive CD4 and CD8 T cells, as measured directly by Ki67 levels, increases with age [36]. Increasing proliferation might be driven by reduced competition from RTEs, whose influx wanes rapidly from adulthood onwards. A nonexclusive possibility is that naive T cells’ intrinsic propensity for proliferation, rather than survival, may increase with cell age in humans. This mechanism has been invoked to explain the relatively sudden loss of naive TCR diversity in the elderly [15].

Whether increasing homeostatic fitness with cell age is beneficial in evolutionary terms is not known. Naive T cells undergo profound changes in their function with host age, showing diminished activation and proliferation in aged mice and humans [56–58]. This development of functional defects appears to be a cell-intrinsic process rather than an effect of the aged environment. Parking of naive CD4 T cells in WT mice for different durations showed that old, longer-lived cells proliferate poorly and produce lower amounts of IL-2 in response to cognate antigen [54], and new naive T cells generated in old mice using bone marrow chimeras exhibit normal function [59, 60]. Despite this loss of function with cell age, it is possible that progressively increasing naive T cells’ ability to persist in the pool helps maintain a sizeable and diverse T-cell repertoire as thymic output wanes. Tuning this persistence via increased survival rather than division is also perhaps desirable because it seems more likely to achieve both stability of cell numbers and maintainance of TCR diversity as replenishment declines. Adaptation as passive accumulation of cells may therefore be an optimal means of making the best of our immune systems as we age. A potential pitfall arises, however, if resource competition plays any additional role in normal naive T-cell homeostasis. In this case, aged and impaired naive cells may actively outcompete younger, more functional ones—an effect that would contribute to the decline in immune responsiveness in the elderly.

## Supporting information

S1 Text Detailed description of models and fitting procedures. (PDF)

S1 Data Data reproduced from Fig 3 of den Braber et al. [8]. (XLSX)

S2 Data Data from busulfan chimera experiments. (XLSX)

S3 Data Data reproduced from Figs 2C and 4E in Tsukamoto et al. [14]. (XLSX)

S1 Fig The density-dependent model fails to explain the replacement kinetics in busulfan chimeras (data provided in S2 Data). (PDF)

S2 Fig Simulating the results of thymectomising busulfan chimeric mice under the different model scenarios. (PDF)

## Abbreviations

AIC: Akaike Information Criterion
BMT: bone marrow transplant
HSC: haematopoetic stem cell
IL-7: interleukin 7
RTE: recent thymic emigrant
TCR: T-cell receptor
TREC: T-cell receptor excision circle
Tx: thymectomised
WT: wild-type
