# Supplementary material for: Age is not just a number: Naive T cells increase their ability to persist in the circulation over time
Source: PLoS Biol. 2018 Apr 11;16(4):e2003949. doi: 10.1371/journal.pbio.2003949 (PMC5894957; doi:10.1371/journal.pbio.2003949)
Supplement: S1 Text — (PDF) [file pbio.2003949.s001.pdf]

S1 Text

Modelling thymic output

We used phenomenological functions to explain changes in the rate of release of new naive cells from the thymus with host age. For the analysis of our busulfan chimeric mice, we assumed that these rates are proportional to the numbers of double-positive ($CD4^+CD8^+$, or DP1) thymocytes, while den Braber *et al.* [1] used the numbers of single positive (SP4 and SP8) compartments from WT mice. We chose the former to exclude effects of contamination of the SP4 and SP8 populations with recirculating mature cells from the periphery.

In both sets of data, all thymic populations appear to decline exponentially from approximately 7 weeks of age:

$$\theta(t) = \theta(0)e^{-\nu t}. \tag{S1}$$

The rate of thymic involution ν was estimated by fitting a linear model to the log-transformed cell counts of DP1 thymocytes. Thymectomy was represented by setting $\theta(t)$ to zero at the time of surgery ($t_0 = 7$ weeks)

$$\theta_{\mathrm{Tx}}(t) = \begin{cases} \theta_0 \, e^{-\nu t} & t \leq t_0 \\ 0 & t > t_0. \end{cases} \tag{S2}$$

To describe changes in thymic output from birth onwards we used the piece-wise function described in den Braber *et al.* [1]. They modelled an exponential increase in the numbers of SP4 and SP8 thymocytes until 7 weeks of age (t_1), a rapid fall between 7 and 8 weeks of age (t_2) and a slower decline thereafter:

$$\theta_{\mathrm{B}}(t) = \begin{cases} \theta_0 \, (1 - e^{-s_1 t}) & t \leq t_1 \\ \theta(t_1) \, e^{-s_2(t-t_1)} & t_1 < t \leq t_2 \\ \theta(t_2) \, e^{-s_3(t-t_2)} & t > t_2. \end{cases} \tag{S3}$$

When modelling data from the busulfan chimeras, we assumed that the total output from the thymus at any time is identical to that in age-matched untreated healthy mice, but is split between donor and host cells according to the chimerism χ at the DP1 stage of thymic development:

$$\begin{aligned} \theta_{\mathrm{donor}}(t) &= \chi\theta(t) \\ \theta_{\mathrm{host}}(t) &= (1-\chi)\theta(t). \end{aligned} \tag{S4}$$

Adaptation model

We solved Eq. 3 in the text using the method of characteristics, with two boundary conditions. One is the constraint that the population density of cells of post-thymic age $a = 0$, $N(t,0)$, at any time t is simply the rate of thymic export at that moment, $\theta(t)$. The second boundary condition is the population density with respect to cell age at time zero, $N(0,a) = g(a)$. Given both, one can then track the fate of the population present at time zero, $N_{\mathrm{init}}(t,a)$ and the fates of cells subsequently exported from thymus, $N_\theta(t,a)$;

$$\begin{aligned} N_{\mathrm{init}}(t,a) &= g(a-t)\exp\left(-\int_0^t \lambda(\tau + a - t)\, d\tau\right), \text{ for } a \geq t \\ N_\theta(t,a) &= \theta(t-a)\exp\left(-\int_0^a \lambda(\tau)\, d\tau\right), \text{ for } a \leq t. \end{aligned} \tag{S5}$$

To connect the adaptation model with observations of naive T cell numbers, we integrated $N_{\text{init}}(t,a) + N_\theta(t,a)$ over cell age to obtain the total population size $N(t)$.

We explored exponential ($\lambda(a) = \lambda_0\, e^{-a/r}$) and sigmoid ($\lambda(a) = \lambda_0/(1+(a/r)^2)$) forms for the net loss rate λ decreasing with cell age. The population density of T cells with respect to cell age at time zero was unknown and we modelled it as $g(a) = e^{pa}\,\theta_0$. The free parameter p could be positive or negative, such that older cells can initially be over- or under-represented compared to younger cells. This definition of $g(a)$ also ensures, for consistency, that $g(0)$ is the rate of output from the thymus at that time, θ_0. This quantity is unknown but can be expressed in terms of $g(a)$ and the initial pool size N_0;

$$\text{Total cell numbers} = \int_0^{t_0} g(a)\,da = \int_0^{t_0} e^{pa}\,\theta_0\,da = N_0 \implies \theta_0 = \frac{N_0 p}{e^{p\,t_a} - 1}, \tag{S6}$$

where the range of possible cell ages at host age t_0 is $0 \le a \le t_0$.

When modelling data from the busulfan chimeras, we considered three subpopulations of naive CD4 or CD8 T cells – a host-derived population generated pre-BMT $N^h_{\text{init}}(t,a)$, the host population post-BMT $N^h_\theta(t,a)$, and the donor population post-BMT $N^d_\theta(t,a)$. Total cell counts are then

$$N_{\text{total}}(t) = \int_0^t \left(N^h_{\text{init}}(t,a) + N^h_\theta(t,a) + N^d_\theta(t,a)\right)\,da. \tag{S7}$$

When modelling the busulfan chimera data, we measured host age from the time of earliest BMT (45 days). In the analysis of the data in den Braber *et al.* [1], time zero was defined either as birth or the time of thymectomy (7 weeks), as appropriate.

Busulfan treatment results in levels of chimerism that vary slightly across mice. To compare replacement across mice, we normalised the fraction of cells in the periphery that were donor-derived to the thymic chimerism χ, defined to be the proportion of thymocytes at the early double-positive (DP1) stage of development that were donor-derived:

$$\text{Normalised donor fraction } f_d = \frac{N^d_\theta(t)}{\chi\, N_{\text{total}}(t)}. \tag{S8}$$

This framework allowed us to combine data from bone marrow transfers made in recipients with different ages. We assumed N_0 and p were free parameters common to all mice that characterised the pool size and age distribution of naive cells at age t_0 = 45 days, the earliest age at BMT in our experiments. For animals that underwent BMT later in life, we used N_0 and p to calculate the predicted naive T cell pool size and age distribution at the time of BMT t_b, by tracking and combining the sizes of (i) the cohort of cells present at t_0 until t_b, which was initially of size N_0 and with age distribution $g(a)$; and (ii) the populations exported from the thymus between t_0 and t_b.

$$\text{Cell age distribution at } t_b = G(a) = \begin{Bmatrix} g(a - (t_b - t_0))\; e^{\int_0^{t-t_0} \lambda(\tau)d\tau} & a \ge t_b - t_0 \\ \theta(t_b - t_0 - a)\; e^{\int_0^{a} \lambda(\tau)d\tau} & a \le t_b - t_0 \end{Bmatrix} \tag{S9}$$

Thymic output as a function of age was modelled as described above, using the forms presented in den Braber *et al.* [1] or as exponential decay when fitting models to busulfan chimera data.

The parameters $N(0)$, p, λ_0, and r were estimated by simultaneously fitting the model to the log-transformed cell counts and logit-transformed normalised donor fractions.

Selection model

We solved Eq. 4 in the text to obtain the distribution of loss rates λ in the naive T cell pool. This distribution is integrated over λ to give total cell numbers in a host of age t:

$$\frac{dN(\lambda,t)}{dt} = \theta(t) f_\theta(\lambda) - \lambda N(\lambda,t),$$

$$N(\lambda,t) = N_0 f_{\text{init}}(\lambda) e^{-\lambda(t-t_0)} + \int_{s=t_0}^{t} \theta(s-t_0) f_\theta(\lambda) e^{-\lambda(t-s)}\, ds, \tag{S10}$$

$$N(t) = \int_0^{\lambda_{\max}} N(\lambda,t)\, d\lambda.$$

Here $f_\theta(\lambda)$ is the distribution of fitnesses of cells emerging from the thymus, assumed to be lognormal with a cutoff $\lambda_{\max}$ chosen to be the 99th percentile of the distribution for any given mean and variance; and $f_{\text{init}}(\lambda)$ is the distribution of fitnesses of cells in the periphery at t_0. Again, thymic output as a function of age was modelled using the forms presented in den Braber *et al.* [1] or as exponential decay when fitting to busulfan chimera data. Donor cell numbers could be calculated based on host age and age at BMT:

$$N_{\text{donor}}(t) = \int_0^{\lambda_{\max}} \left(\int_{s=t_b}^{t} \theta(s-t_0) f_\theta(\lambda)\, e^{-\lambda(t-s)}\, ds \; + N_0\, f_{\text{init}}(\lambda) e^{-\lambda(t-t_0)} \right) d\lambda,$$

$$f_d = \frac{N_{\text{donor}}(\lambda,t)}{\chi\; N_{\text{total}}(\lambda,t)}. \tag{S11}$$

The selection model has six unknowns (N_0, θ_0, μ_θ, σ_θ, μ_{init} and σ_{init}), which, as before, were estimated by simultaneously fitting the model to the log-transformed cell counts and logit-transformed normalised donor fractions.

Incumbent model

In this model, proposed in [2], the peripheral naive CD4 and CD8 T cell populations are assumed to be heterogeneous, each consisting of a 'displaceable' subset continuously supplemented from the thymus and an 'incumbent' subpopulation established early in life that is nearly self-renewing (*i.e.*, with low net loss rate λ_i):

$$\begin{aligned} \frac{dN_{\text{donor}}}{dt} &= \chi\, \theta_0\, e^{-\nu t} - \lambda\, N_{\text{donor}}(t) \\ \frac{dN_{\text{host}}}{dt} &= (1-\chi)\, \theta_0\, e^{-\nu t} - \lambda\, N_{\text{host}}(t) \\ \frac{dI}{dt} &= -\lambda_i I(t). \end{aligned} \tag{S12}$$

As described in ref. [2], we solved these ODEs to obtain total cell counts $N(t)$, with initial condition $N(0) - I(0) = N_{\text{donor}}(0) + N_{\text{host}}(0)$, where $N_{\text{donor}}(0)$, $N_{\text{host}}(0)$ and $I(0)$ are the numbers of donor, host and incumbent cells present at t_0, respectively. In the incumbent model t_0 corresponds to the time at which host-donor chimerism has stabilised at all stages of thymic development, estimated to be 6 weeks post bone marrow transfer, and not the time of earliest bone marrow transfer as in the models above. We accounted for different ages at BMT by tracking changes in the donor population $N_{\text{donor}}(0)$ with host age. The initial cell counts

and normalised donor fractions in mice that underwent BMT later in life (age t_b) are then

$$N(t_b) = \frac{\theta(t-t_0)\ (e^{(\lambda-\nu)(t_b-t_0)}-1) + (\lambda-\nu)\,(N(0)-I(0)) + (\lambda-\nu)\ I(0)\,e^{(\lambda-\lambda_i)(t_b-t_0)}}{(\lambda-\nu)\ e^{\lambda(t_b-t_0)}}$$

$$f_d(t_b) = \frac{N(0)\,f_d(0)\,e^{-\nu(t_b-t_0)}}{N(t_b)}, \tag{S13}$$

and the total cell counts and normalised donor fraction as functions of host age are

$$N(t) = \frac{\theta(t-t_0)\ e^{-\nu(t_b-t_0)}\ (e^{(\lambda-\nu)(t-t_b)}-1) + (\lambda-\nu)\,(N(t_b)-I(t_b)) + (\lambda-\nu)\ I(t_b)\,e^{(\lambda-\lambda_i)(t-t_b)}}{(\lambda-\nu)\ e^{\lambda(t-t_b)}}$$

$$f_d(t) = \frac{\theta(t-t_0)\ e^{-\nu(t_b-t_0)}\ (e^{(\lambda-\nu)(t-t_b)}-1) + (\lambda-\nu)\,N(t_b)\,f_d(t_b)}{\theta(t-t_0)\ (e^{(\lambda-\nu)(t-t_b)}-1) + (\lambda-\nu)\,(N(t_b)-I(t_b)) + (\lambda-\nu)\ I(t_b)\,e^{(\lambda-\lambda_i)\,(t-t_b)}}. \tag{S14}$$

For fits to data from den Braber *et al.* [1], θ and θ_{Tx} (Equations S1 and S2) were used for WT and Tx mice respectively and N_{donor} was set to zero. In ref. [2] the incumbent loss rate was found to be statistically indistinguishable from zero and so we set $\lambda_i = 0$.

The incumbent model has four free parameters ($N(0)$, θ_0, $I(0)$ and λ) when fitted to the log-transformed cell counts of naive T cells when using data from den Braber *et al.* [1]. Fitting the data from busulfan chimeras required an additional free parameter $f_d(0)$, the normalised donor fraction at t_0.

Statistical Analyses

Models were fitted to data in *R* using optim with the Nelder-Mead algorithm, using the log likelihood as the objective function. When fitting models simultaneously to multiple datasets, we accommodated different degrees of (unknown) noise or measurement error by calculating the maximum likelihood estimate of the error variance within each dataset and then maximising the joint profile likelihood with respect to the model parameters, as described in [2]. Bootstrapped 95% confidence intervals on parameter estimates were obtained by re-sampling residuals 1000 times. Model selection was performed using the Akaike Information Criterion [3].

References

[1] den Braber I, Mugwagwa T, Vrisekoop N, Westera L, Mögling R, de Boer AB, et al. Maintenance of Peripheral Naive T Cells Is Sustained by Thymus Output in Mice but Not Humans. Immunity. 2012;36(2):288–297. doi:10.1016/j.immuni.2012.02.006.

[2] Hogan T, Gossel G, Yates AJ, Seddon B. Temporal Fate Mapping Reveals Age-Linked Heterogeneity in Naive T Lymphocytes in Mice. Proc Natl Acad Sci USA. 2015;112(50):E6917–6926. doi:10.1073/pnas.1517246112.

[3] Akaike H. A new look at the statistical model identification. IEEE Transactions on Automatic Control. 1974;19(6):716–723.
