# Supplementary material for: Age is not just a number: Naive T cells increase their ability to persist in the circulation over time
Source: PLoS Biol. 2018 Apr 11;16(4):e2003949. doi: 10.1371/journal.pbio.2003949 (PMC5894957; doi:10.1371/journal.pbio.2003949)
Supplement: S1 Fig — (PDF) [file pbio.2003949.s005.pdf]

S1 Figure

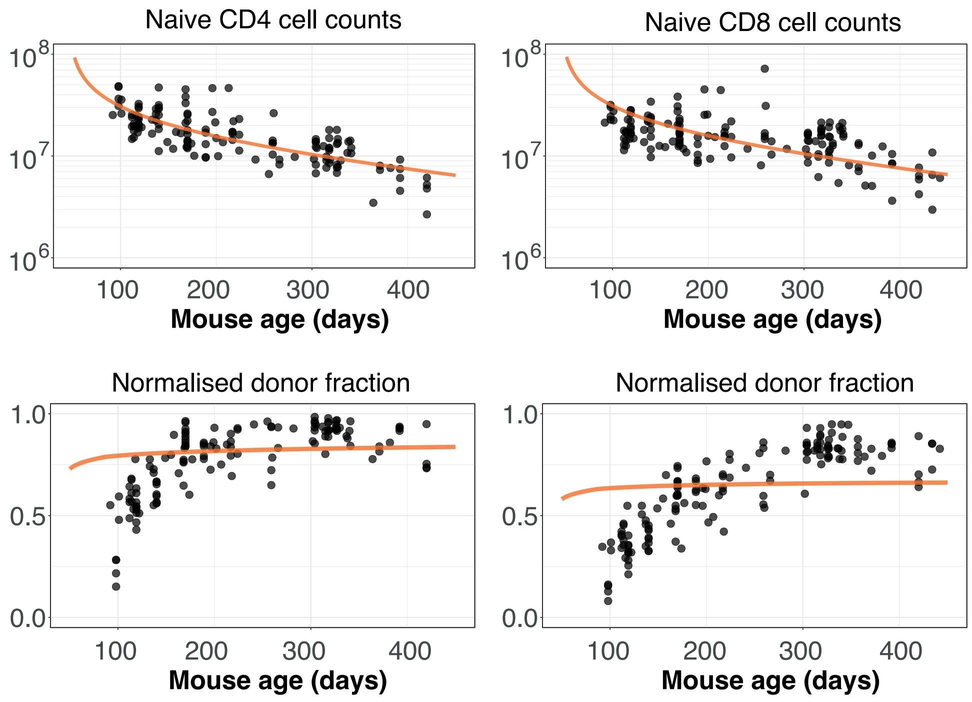

Figure S1: **The density-dependent model fails to explain the replacement kinetics in busulfan chimeras. Data are as in Fig 3 in the main text.**
