# Supplementary material for: Age is not just a number: Naive T cells increase their ability to persist in the circulation over time
Source: PLoS Biol. 2018 Apr 11;16(4):e2003949. doi: 10.1371/journal.pbio.2003949 (PMC5894957; doi:10.1371/journal.pbio.2003949)
Supplement: S2 Fig — (PDF) [file pbio.2003949.s006.pdf]

S2 Figure

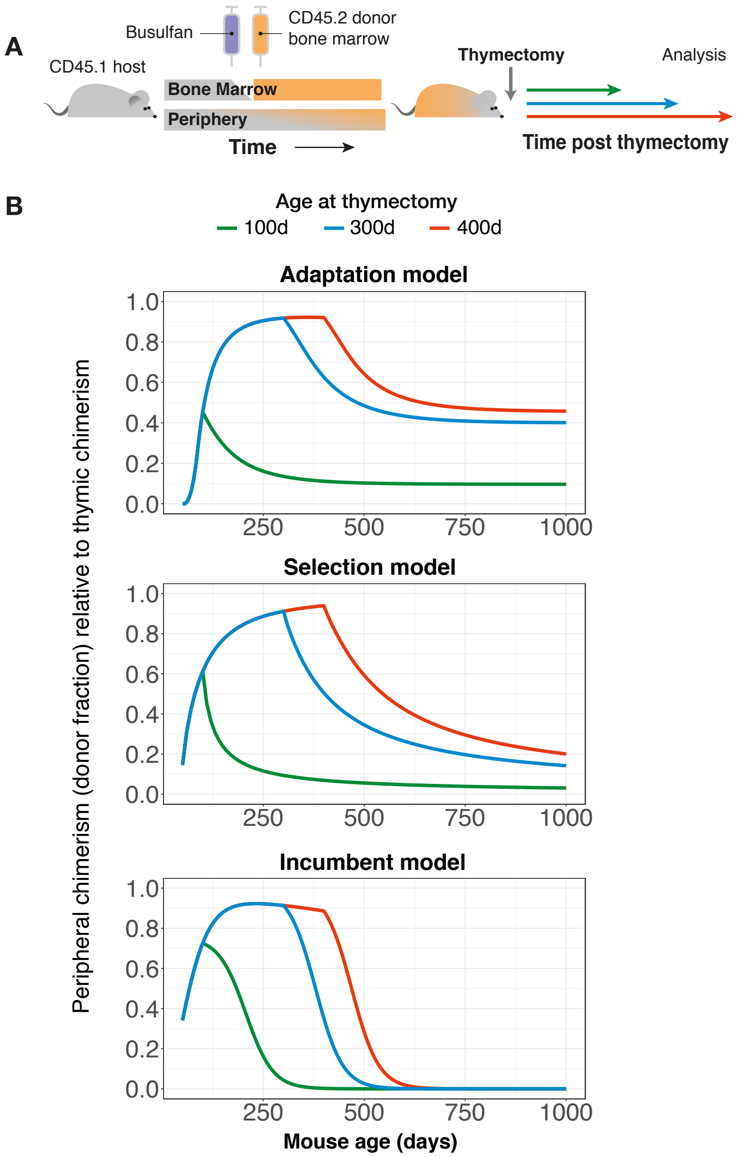

Figure S2: **Predicted kinetics of the normalised donor fractions post-thymectomy in busulfan chimeras.** **(A)** Strategy for the proposed experiment. **(B)** Kinetics of peripheral chimerism normalised to thymic chimerism (f_{d}), obtained by simulating thymectomy at different times post-BMT in busulfan-chimeric mice, and using parameters estimated from the busulfan chimera data presented in this study. Top panel: The adaptation model predicts that f_{d} drops gradually over time post-thymectomy to a steady state value, because both donor and host cells consistently gain fitness with cell age, and in this model the maximal fitness corresponds to a net loss rate of zero ($\lambda(a) \to 0$ for large a). Middle panel: In the selection model, the predicted f_{d} declines consistently without reaching a steady state value. There is continual enrichment for cells drawn from the tail of the distribution of fitnesses generated during development. Host cells will therefore always be more enriched for long-lived cells than the donor population, and so the donor fraction will never equilibrate. Further, mice thymectomised early in life will have had relatively little time to preferentially accumulate high-fitness cells through selection. We therefore predict that these mice will show a faster initial loss of naive cells (green line) than animals thymectomised later in life (blue and red lines). Bottom panel: Predictions from the incumbent model suggest that f_{d} post-thymectomy drops to zero, since all donor cells are assumed to be displaceable and are eventually lost from the periphery.
